# Antimicrobial 3D printed implants for periprosthetic joint infections

**DOI:** 10.1007/s13346-025-01934-5

**Published:** 2025-08-14

**Authors:** Iván Yuste, Francis C. Luciano, Carmina Rodríguez, Bianca I. Ramirez, Chrysi Rapti, Brayan J. Anaya, Aikaterini Lalatsa, Almudena Ribed-Sánchez, Pablo Sanz-Ruiz, Elena González-Burgos, Dolores R. Serrano

**Affiliations:** 1 Pharmaceutics and Food Technology Department, Faculty of Pharmacy, Universidad Complutense de Madrid, Plaza Ramón y Cajal s/n, Madrid, 28040 Spain; 2 Department of Microbiology and Parasitology, Faculty of Pharmacy, Universidad Complutense de Madrid (UCM), Madrid, Spain; 3 School of Pharmacy and Biomedical Sciences, Robertson Wing, University of Strathclyde, 161, Cathedral Street, Glasgow, G4 0RE Scotland, UK; 4 Hospital Pharmacy Unit, Hospital General Universitario, Doctor Esquerdo 46, Gregorio Marañón, Madrid, 28029 Spain; 5 Orthopaedic and Trauma Department, Hospital General Universitario, Gregorio Marañón, Doctor Esquerdo 46, Madrid, 28029 Spain; 6 Department of Surgery, Faculty of Medicine, Universidad Complutense de Madrid (UCM), Madrid, Spain; 7 Department of Pharmacology, Pharmacognosy and Botany, Faculty of Pharmacy, Universidad Complutense de Madrid (UCM), Madrid, Spain; 8 Faculty of Pharmacy, Instituto Universitario de Farmacia Industrial, Universidad Complutense de Madrid, Madrid, 28040 Spain

**Keywords:** 3D printing, Implant, Bone cement, PJIs, Infection, Prosthesis, Hip, Knee, Amphotericin B, Vancomycin

## Abstract

Periprosthetic joint infections (PJIs) remain a serious complication following hip and knee arthroplasty, affecting 1–5% of patients in developed countries and posing significant challenges to patients, clinicians, and healthcare systems. Conventional prophylactic strategies, such as antibiotic-loaded bone cement, suffer from limited post-implantation drug release and potential compromise of mechanical integrity. To overcome these limitations, we developed a personalized, 3D-printed implant designed to integrate with the acetabular component of joint prostheses. These implants deliver either monotherapy or a combination of amphotericin B (AmB) and vancomycin (VAN), targeting both fungal and bacterial pathogens. Fabricated via fused deposition modeling using a biocompatible polyvinyl alcohol–polyethylene glycol (PVA–PEG) matrix, the implants enable passive drug loading and rapid adhesion to prosthetic surfaces within 60 s, minimizing operative time. In vitro testing confirmed broad-spectrum antimicrobial activity against *Candida* spp. (*C. albicans*, *C. parapsilosis*, *C. glabrata*, *C. krusei*) and *Staphylococcus* spp. (*S. aureus*, *S. epidermidis*). VAN was released rapidly, while AmB exhibited sustained release for up to 10 h, with both maintaining saturation solubility for 48 h. Notably, AmB-loaded implants showed five-fold lower hemolytic toxicity compared to free drug. These results highlight the potential of 3D-printed, drug-eluting implants as a clinically viable solution for the prevention and early treatment of PJIs.

**Graphical abstract:**

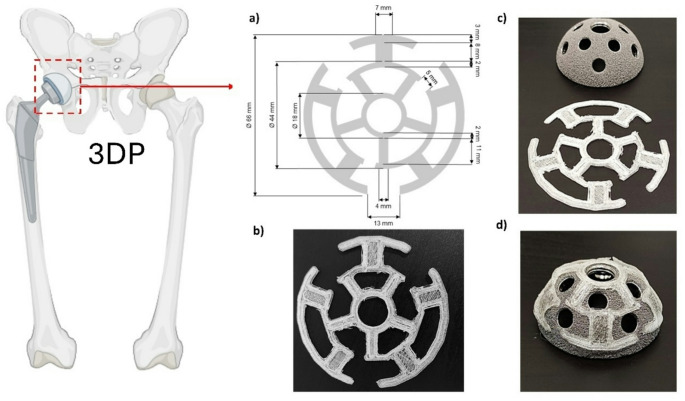

**Supplementary Information:**

The online version contains supplementary material available at 10.1007/s13346-025-01934-5.

## Introduction

The total number of joint arthroplasties, specifically hip and knee replacements, has experienced a remarkable increase since 2000 in most developed countries as described by the Organization for Economic Co-operation and Development (OECD). Between 2000 and 2015, the rate of hip replacement increased by 30% (166 operations over 100,000 population) and by 50% in knee replacement (126 operations over 100,000 population) [1, 2]. Moreover, it is estimated that there will be a 100% increase in hip arthroplasty from 2015 to 2050 and a 43% in knee arthroplasty from 2022 to 2050 [3].

Currently, the most common treatments used to prevent PJIs are (i) the irrigation of the prosthesis with an antibiotic solution, (ii) the pre and post-administration of oral and intravenous antibiotics for prolonged periods, and (iii) the use of polymethylmethacrylate types of bone cement (PMMAs) loaded with antibiotics after removing the prosthesis and cleaning the surrounding tissues, which elution rates range from 4 to 17%. When prostheses are irrigated with antibiotics, the local concentration drops very fast, being insufficient to prevent infections. The administration of oral or intravenous antibiotics for long periods can lead to systemic adverse effects, and low local concentrations at the joint level are achieved [4, 5].

There are several limitations with antibiotic-loaded cements. First, drug delivery is insufficient after surgery, especially for lipophilic drugs, resulting in a very low antibiotic release and, therefore, a high risk of resistance. The second limitation is that the biomechanical properties of the joint can be altered once the antibiotic is loaded in the cement; the compressive strength of the cement can be reduced up to 40% after elution, and consequently, the life of the patient can be compromised [6, 7]. Currently, a few already-marketed PMMAs contain one antibiotic, such as gentamicin, like Palacos^®^ R or Simplex bone cement. However, no antifungal loaded-PMMAs cement is commercially available, and cements loaded with antibiotic combinations are still in clinical trials to improve the activity of the commercially available ones, but drug interaction remains a major challenge still unsolved [8]. There is an unmet clinical need to find innovative solutions to reduce the risk of PJIs [9]. PJIs are most likely to originate during or immediately after surgery, making the initial 24-hour postoperative period a critical window for prophylactic intervention. Bacterial contamination of the surgical site often occurs intraoperatively or within the first few hours postoperatively, with pathogens adhering to implant surfaces and initiating biofilm formation within 6–24 h its surface [10–12]. Once a biofilm is established, treatment becomes significantly more difficult due to increased antimicrobial resistance and host immune evasion. As such, delivering high local concentrations of antimicrobials during this early phase is essential for preventing infection and avoiding the need for revision surgery. While bacterial infections remain the most common cause of PJIs, the clinical burden of fungal PJIs, though rare (estimated at < 1% of all PJIs), is disproportionately high due to delayed diagnosis, limited treatment options, and poor outcomes [13, 14]. *Candida* spp., in particular, are increasingly implicated in immunocompromised or high-risk patients and have been associated with biofilm-mediated infections on prosthetic surfaces. Therefore, there is a growing need for short-term, broad-spectrum drug delivery systems capable of preventing both bacterial and fungal colonization during the immediate postoperative period [15, 16].

The hypothesis underpinning this work is that a controlled drug-release implant for parenteral administration, well-adapted to the prosthesis shape with a sustained drug-release profile, would be suitable to prevent and treat PJIs during the first 24 h after implantation. In this work, we have advanced the state-of-the-art by 3D printing implants designed and manufactured a bioinspired-shaped implant adapted to the surface of the acetabular component of a hip prosthesis with millimetric dimensions for adequate spacing to enable osteointegration. Implants were loaded with an antifungal, an antibacterial, or a combination of both. Amphotericin B (AmB) was selected as the model antifungal as it is the gold standard for the treatment of systemic fungal infections [17, 18]. Vancomycin (VAN) was selected as a broad-spectrum antibacterial agent commonly employed in clinical practice to treat PJIs [19]. The physicochemical and biological performance of the 3D printed implants was investigated to support their clinical translation.

## Materials and methods

### Materials

Vancomycin hydrochloride (VAN) with a purity of ≥ 85% was supplied by the Gregorio Marañón Hospital (Pfizer, Madrid, Spain) and AmB was purchased from Kemprotec Limited (> 96%, Cumbria, UK). Hydrosupport© filament was purchased from 3D DomFuel (Donegal, Ireland). Human albumin (Albutein^®^ 20%) was purchased from Grifols^®^ (Barcelona, Spain). Commercial 6 mm disks of VAN (30 µg) and AmB (10 µg) were purchased from Neo-Sensitabs (Rosco, Denmark). Salts (ACS grade) and solvents (HPLC-grade) were purchased from Proquinorte (Madrid, Spain). All other chemicals and solvents were at least of ACS reagent grade and were used without further purification.

### Design, engineering, and optimisation of the 3D printed implant

**Fig. 1:**
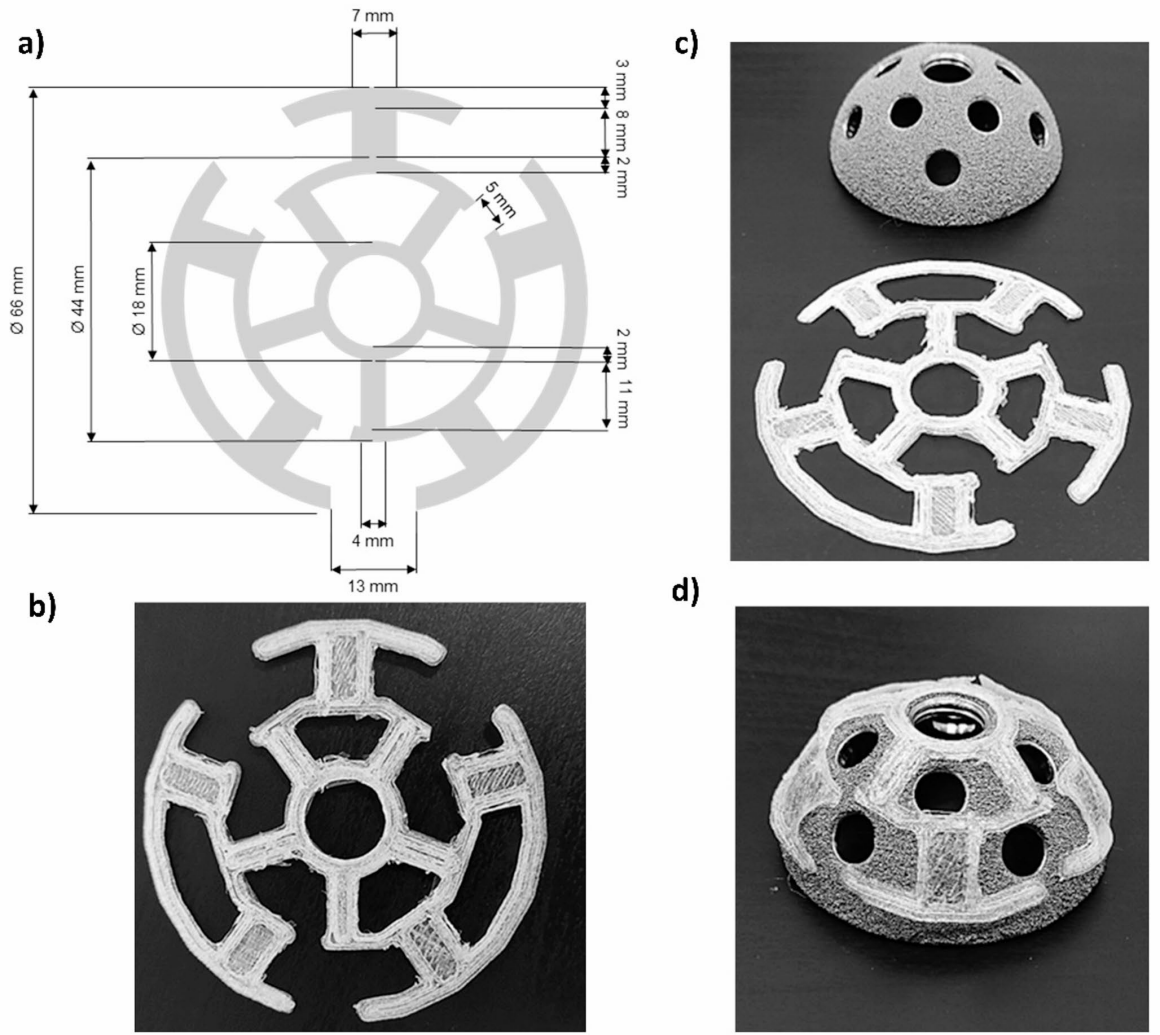
3D printed implant adapted to the acetabular component morphology. Key: (**a**) Design and dimensions of the implant, (**b**) 3D printed implant; (**c**) Dimensions of 3DP implant and acetabular component, and (**d**) 3DP implant adhered onto the surface of the cup

The dimensions of the acetabular surface were carefully measured with an electronic digital caliper (resolution: 0.1 mm, accuracy: ± 0.2 mm). This aided the design of the parenteral implant in a spider-web shape to improve adhesion and be adapted to the convex morphology of the acetabular cup using Tinkercad (Autodesk, Ohio, USA). The implant was circular with a diameter of 64 mm (all dimensions are described in full in Fig. 1).

Implants were printed with a fused deposition model (FDM) Hyrel 3D Engine SR printer (Hyrel, Georgia, USA) equipped with an MK1-250 extruder head. The Hydrosupport^®^ filament, consisting of partially saponified polyvinyl alcohol (PVA) (> 94.8%) with polyethylene glycol (PEG) fragments (3D DomFuel, Donegal, Ireland), was selected due to its hydrophilic and biocompatible nature. The filament was extruded using a temperature ranging from 210 °C to 215 °C and the platform temperature was kept at 70 °C to ensure good adhesion of the 3D implant to the platform. The nozzle diameter was 0.5 mm and the layer height was 0.25 mm. The 100% infill with a rectilinear pattern was selected with a printing speed of 60 mm/s, and a travel speed set at 30 mm/s.

After printing, the implant was immersed in a solvent mixture containing the drug to be loaded by passive diffusion. The drug loading process by passive diffusion was optimised for AmB using Design of Experiment (DoE) studies utilising Design-Expert 10 software (Stat-Ease Inc., Minneapolis, USA). A 2³ factorial design was carried out. Three variables were studied at 2 levels each: (i) height of the implant (1–2 mm), (ii) solvent mixture utilised for drug solubilisation (Ethanol: DMSO, 75:25 or 85:15 v/v), (iii) the concentration of AmB in the solution (2.5 or 5 mg ml^− 1^). The optimal conditions were kept constant for the loading of vancomycin and also for the loaded of both drugs simultaneously. The rationale behind this is the fact that AmB is a poorly water-soluble drug while vancomycin is a salt with much greater water solubility.

### Evaluation of drug loading kinetics across the 3D-printed implant

The kinetic rate of passive diffusion of VAN, AmB, or both to the 3D-printed implant was investigated (*n* = 3). First, 150 mg of each active ingredient was dissolved in 7.5 ml of DMSO, vortexed, and sonicated for 15 min, before the solution was added dropwise to 22.5 ml of ethanol. 3D printed implants were then placed on special supports designed in Tinkercad and printed using a Creamelt polymer (cyclic olefin copolymer) (Fig. S1) to keep the implant fully in contact with the solvent media (ethanol-DMSO), avoiding adhesion to the bottom of the petri dish. The solvent mixture was added to the Petri dish, covering the surface of the implant (20 ml). At certain time points (0, 1, 2, 3, 4, and 5 h), a fragment of the immersed implant (10 mg) was cut, withdrawn, and dissolved in 10 ml of deionized water. From this solution, 750 µl were withdrawn and mixed with 750 µl of the mobile phase. The amount of drug diffused per unit of area of the implant was quantified by HPLC (Varian Prostar 230 Solvent Delivery Module, Varian Prostar autosampler 410, and Varian Prostar 310 UV-visible detector (Varian, Palo Alto, CA, USA)).

AmB was separated on a Thermo Hypersil-Keystone BDS column (200 × 4.6 mm, 5 μm). The mobile phase consisted of acetonitrile: glacial acetic acid: water (52:4.3:43.7 v/v), which was pumped at a flow rate of 1 mL/min. The sample injection volume was set at 40 µL, the column temperature was maintained at 25 °C, and the detector was set at 406 nm [20]. VAN was analysed using a Nucleosil C18 column (250 × 4.6 mm, 5 μm) [21, 22] and a mobile phase consisting of 50 mM ammonium phosphate: acetonitrile (92:8, v: v, pH of 2.2) which was pumped at a flow rate of 0.7 mL/min. The injection volume of the sample was 40 µL, and the column temperature was maintained at 25 °C with the detector set at 205 nm [23].

### Compression strength and adhesiveness

The compression strength and adhesiveness of AmB and vancomycin-loaded implants were evaluated in triplicate using aTexture Analyzer TA.XT Plus C (Stable Micro Systems Ltd., Godalming, UK). Results were compared with blank unloaded implants. The test was applied to implants in a dry state, but also after immersion for 60 s in deionized water. The implant was mounted onto the center of the base of the texture analyzer. A 1/2” Ø Cylinder Probe (p/0.5R) was moved down at a constant speed of 0.5 mm/s. Once the probe was in contact with the implant, the compression force was recorded to displace the probe down by 0.1 mm. Afterward, the probe was detached at a post-test speed of 1 mm/s, and the adhesiveness to the probe was evaluated. Data was collected at a rate of 200 points per second (PPS). The Exponent software (version 8.0.14.0, Stable Micro Systems, Godalming, UK) was used for the test [24].

A manual adhesiveness test was also performed to mimic surgical conditions. After printing, loaded, and unloaded 3DP implants (*n* = 3) were wetted in 20 ml of deionized water at different times (15, 30, 45, 60, 90, and 120 s). Then, implants were placed on the surface of the acetabular component of the prosthesis using tweezers. Manual pressure was exerted on the surface of the implants to ensure intimate contact with the prosthesis for 5 s. Three levels of adhesion were established: (3) when all the surface of the implant has fully adhered to the acetabular component surface and did not detach when turned upside down, (2) when the implant has partially adhered to the acetabular component and detached partially when turned upside down, and (1) low when the implant was fully detached from the surface of the prosthesis.

### Morphology characterization with scanning electron microscopy (SEM)

A section of the loaded and unloaded implants was cut with a scalpel and placed on carbon-coated stubs that were sputter-coated with gold for 120 s (QUORUM Q150R S Mussashime, Japan) to help conductivity. The morphology of the 3DP implant was characterized by SEM (JEOL 6335 F, Mussashime, Japan) with a voltage of 20 KV at different magnifications.

### Sterilization by UV light

Loaded 3D-printed implants with VAN and/or AmB were dried in the open air for 24 h, stored in a transparent airtight bag (to avoid contamination after sterilization), and placed under UV light for 20 min, exposing each side of the implants for 10 min (*n* = 3). After that time, the implants were turned over and exposed to UV light for an additional 10 min. Once the sterilization process was complete, small fragments of the sterilized implants were collected, and the drug content was analyzed by HPLC. Drug content was evaluated before and after exposure to UV light. After sterilization, implants were placed on Mueller-Hinton Broth (MHB) plates for 48 h to evaluate the presence or absence of microorganisms and count the colony-forming units.

### Drug release

Implants of 1.135 ± 0,071 g loaded with VAN (5 mg), AmB (2.5 mg), and both active ingredients were immersed for 90–120 s in deionized water (20 ml) before adhesion to the acetabular cup (*n* = 3). The prosthesis with the 3D implant adhered to its surface was placed facing down on a 3D printed concave receptacle adapted to the specific dimensions of the prosthesis, mimicking the joint space with a maximum capacity of 10 ml (Fig. S2). The media mimicking the physiological conditions consisted of PBS (1X, pH 7.4) with 20% human albumin (Grifols^®^^,^ Barcelona, Spain) (80:20 v/v). The addition of albumin to the medium was important for high protein-bound drugs such as AmB (> 95%). The receptacles were covered with parafilm and were kept at 37 °C for 48 h. At different times (0, 0.5, 1, 3, 5, 7, 9, 12, 24, and 48 h), samples (750 µl) were withdrawn and diluted with methanol (1:1 v/v) for analysis, while the media volume was replaced with 750 µl at each time point to maintain sink conditions.

Collected samples were frozen for 24 h at -20 °C to ensure maximal precipitation of proteins (e.g., albumin) before centrifugation (10,000 rpm, 15 min). The supernatant (350 µl) was diluted (1:3 v/v) with methanol (1,050 µl), followed by centrifugation (10,000 rpm for 15 min). The supernatant was analysed by HPLC to quantify drug release from the 3D-printed implants at different time points.

### Haemolysis studies

Red blood cells (RBCs) were obtained by centrifugation of blood collected from a healthy 27-year-old male (5.48 × 10^6^ RBC/ml) in K_2_-EDTA-coated Vacutainer^®^ tubes (Becton Dickinson and Co., New Jersey, USA) for 5 min at 1000 g. Haemolysis studies were performed as previously described in triplicate [25].

### Antimicrobial assays

#### Antifungal in vitro assay

The antifungal activity of AmB-loaded implants against four different strains of the pathogenic dimorphic yeast *Candida* spp. (*Candida albicans* CECT 1394, *Candida parapsilosis* 57744, *Candida glabrata* 60750, and *Candida krusei* 1068) was evaluated by agar diffusion assay as previously described in quintuplicate [26, 27]. Implants of 13 mm in diameter were printed, loaded with 200 µg of AmB, and placed in the center of the agar plates. The size of the implant was sufficiently reduced to fit within the agar plate and allow the formation of inhibition halos. Discs loaded with AmB dissolved in DMSO (10 µg in 20 µL) and commercial discs of AmB (10 µg, diameter: 6 mm, Neo-Sensitabs, Rosco, Denmark) were used as a positive control.

#### Antibacterial in vitro assay

The antibacterial activity of implants loaded with VAN was tested against *Staphylococcus epidermidis* (ATCC 12228) and *Staphylococcus aureus* (ATCC 23235) in quintuplicate. The antimicrobial activity was tested by diffusion assay in Kirby-Bauer agar as previously described [28, 29]. Implants of 13 mm in diameter were printed, loaded with 400 µg of VAN, and placed in the center of the agar plates. Commercial discs of vancomycin (30 µg, diameter: 6 mm, Neo-Sensitabs, Rosco, Denmark) were used as a positive control. DMSO, unloaded implants, implants impregnated just with DMSO, and 6 mm diameter discs loaded with DMSO were also used as controls. Isolates were classified as vancomycin susceptible (S) when the zone of inhibition was greater than 15 mm according to the CLSI guidelines [30].

#### Minimal fungicide concentration (MFC), minimal bactericidal concentration (MBC), and minimum inhibitory concentration (MIC)

MFC, MBC, and MIC were evaluated as previously described in triplicate [29]. Cultures were prepared by picking a single colony from a 24-h Mueller Hinton Agar (MHA) plate and resuspending with 0.9% sterile physiological saline solution for *Candida* spp. and MHB for *Staphylococcus* spp. to achieve absorbance of 0.1 at 600 nm, corresponding to 1 × 10^6^ colony forming units (CFU)/ml for *Candida* spp. and 1 × 10^8^ CFU/ml for *Staphylococcus* spp., based on the 0.5 McFarland standard. The suspension was further diluted with Mueller Hinton Broth (MHB) up to a concentration of 4 × 10^5^ CFU/ml.

Fragments of the loaded 3D printed implant were dissolved in 1,950 µl of MHB to obtain concentrations ranging from 0.06 to 2 µg/ml of AmB and concentrations ranging from 2 to 64 µg/ml of VAN. The inoculum (50 µl) containing 2 × 10^4^ CFU was added to each solution, except in the medium-growth control tubes. As a positive control for fungal and bacterial growth, just the inoculum was added to MHB. Tubes were incubated at 37 ˚C under shaking at 180 rpm for 24 h. The turbidity of the tubes was visually inspected to determine the MIC. Additionally, to calculate the MFC, 5 µL from each tube was cultured on Sabouraud agar plates (*Candida* spp.) or MHA (*Staphylococcus* spp.), which were further incubated for 24 h at 37 ˚C (drop plate method) [31]. The MFC was determined as the lowest concentration with the absence of colony growth.

### Statistical analysis

Minitab 16 (Minitab Ltd., Coventry, UK) was used for the statistical analysis. One-way ANOVA test was performed for the haemolysis, antifungal, and antibacterial in vitro assays, and T-Test for UV sterilization, considering *p*-values for statistical significance to be below 0.05.

## Results

### Design of the 3D-printed implant

The implant was designed to be adapted to the convex morphology of the acetabular cup, with empty spaces to allow screw placement during the surgical process. The design was comprised of three concentric circles with outer diameters of 66, 44, and 18 mm, a 2 mm width for the inner and middle circles (44 and 18 mm), and 3 mm for the outer one (66 mm). The first and second circles were interconnected with branches of 8 × 7 mm, while the second and third circles were connected by arms of 11 × 4 mm. The outer and middle circles presented gaps of 13- and 5-mm distance for better adhesion to the acetabular component (Fig. 1a). The geometrical design shown in Fig. 1a was sliced and exported as a.stl file before printing, and the sliced file was transferred to the FDM printer. The 3DP implant geometry matched the.stl file accurately (Fig. 1b).

### Optimization of the implant height and loading process

**Fig. 2:**
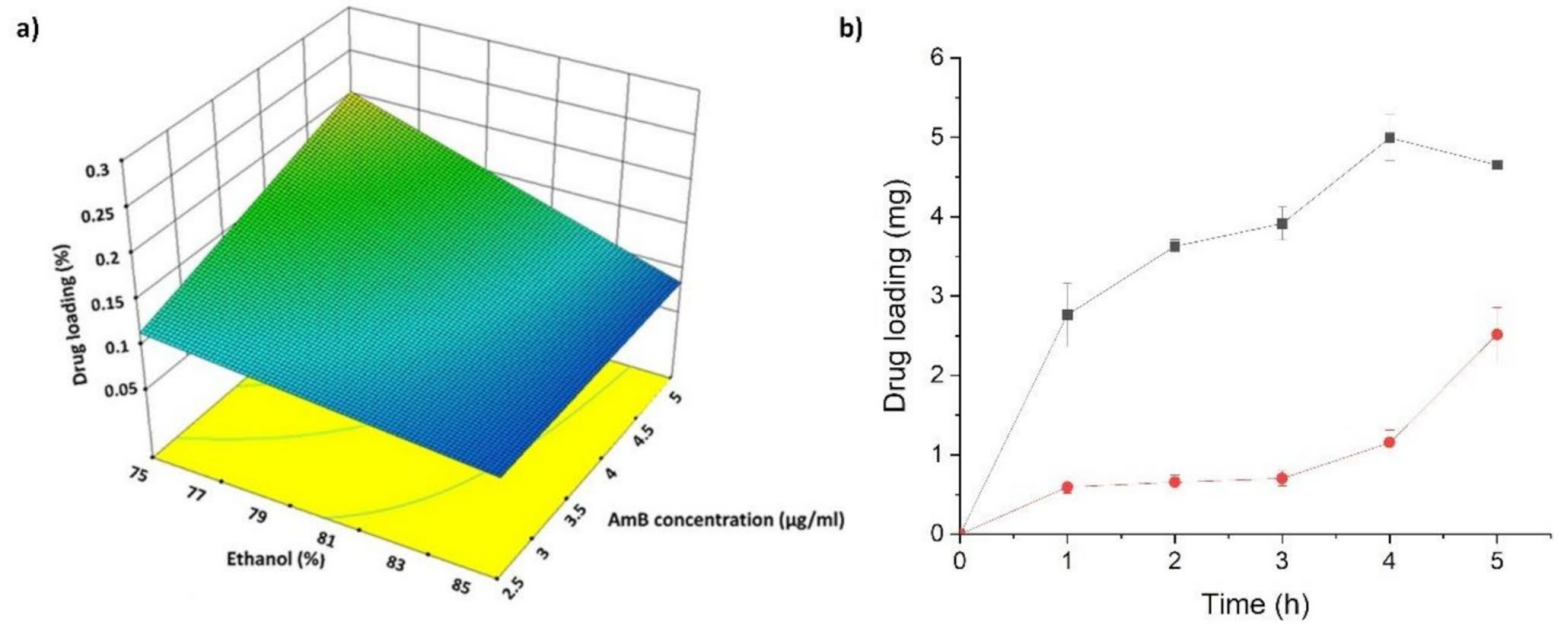
3DP implant optimization and drug loading kinetics. Key: (**a**) Quality by design and optimization of AmB-loaded 3D printed implants; (**b**) Drug loading kinetics of VAN (-■-) and AmB () within the 3DP implants

Once the X and Y dimensions of the implant were established, the next step was to optimize the height, which was included as a variable in the DoE. In addition to the height of the 3DP implant, the effect of solvent and drug concentration during the loading process was also evaluated. The height of the implant that allowed the best drug loading was 1 mm. The passive diffusion drug loading process was more successful when ethanol: DMSO (3:1 v/v) was employed as the solvent mixture, and the active ingredient (AmB in this case) was available at a concentration of 5 mg ml^− 1^ (Table S1). The highest drug loading for AmB achieved was 0.2% (Fig. 2a), while double that was achieved for VAN (0.4%).

### Evaluation of the drug loading kinetics

Drug loading was significantly different for each active ingredient. For VAN, the drug loading followed a linear kinetic rate of 1.114 mg/h (R^2^ = 0.8715) for up to 4 h. Longer diffusion times did not lead to higher drug loading. In the case of AmB, the loading kinetics were bilinear, with a loading rate of 0.218 mg/h (R^2^ = 0.7275) during the first three hours of contact with the solvent and 0.903 mg/h (R^2^ = 0.9217) from 3 to 5 h (Fig. 2b). The lower drug loading of AmB may be due to its low solubility in water, which can lead to precipitation, and the change in the loading kinetics may be due to the pore formation in the implant [32]. The maximum drug loading time was achieved at 5 h. Longer periods were not evaluated due to the lack of rigidity of the structure and considerable loss of morphology upon immersion in the solvent mixture.

### Compression strength and adhesiveness

**Fig. 3:**
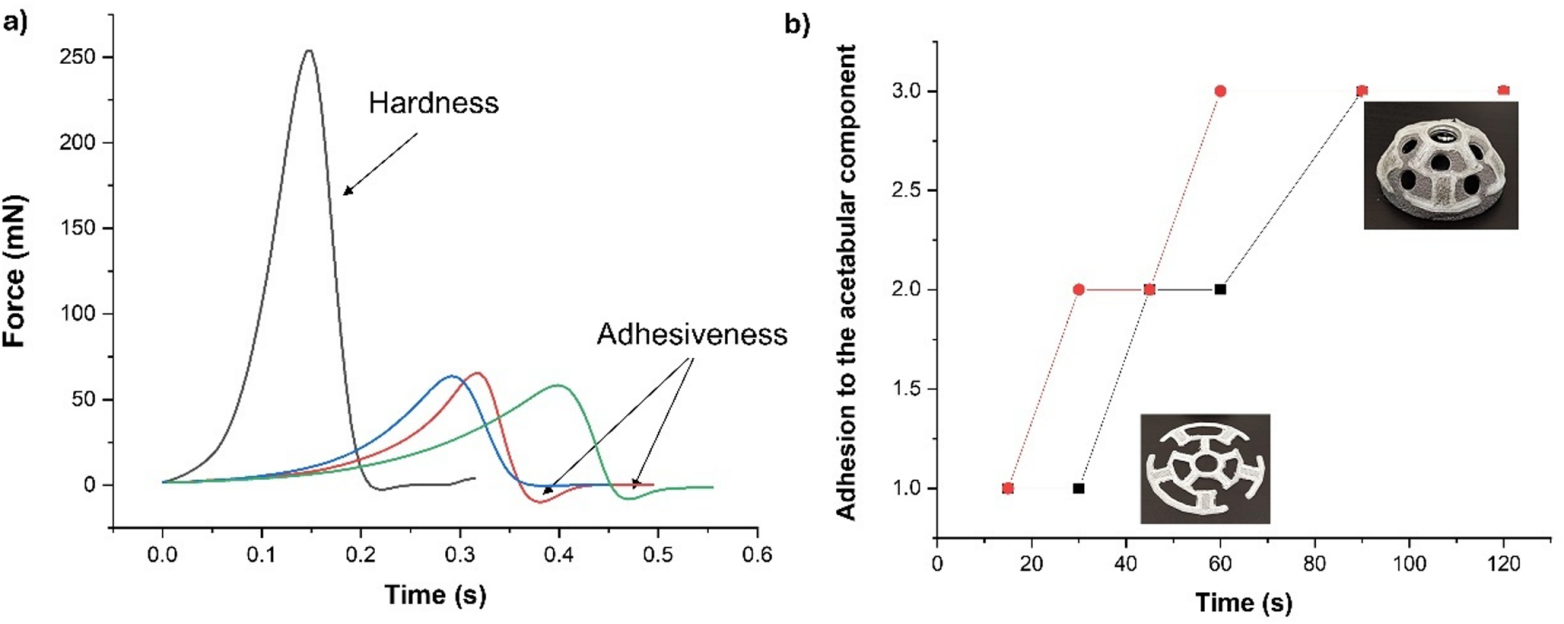
Compression strength and adhesiveness of the 3D printed implants. **a**) Evaluation using the texture analyser by applying a 50 mN force. Key: (black) Dry Blank implant, (red)Wet blank implant; (blue) AmB and Vancomycin -loaded dry implant and (green Wet AmB and Vancomycin -loaded dry implant; **b**) Manual pressure exerted on the loaded and blank implants on the surface of the acetabular component. Key: 1 refers to poor adhesion and 3 to excellent adhesion to the acetabular component

Dry unloaded blank implants showed a significantly higher (5-fold) hardness compared to wet unloaded implants (Fig. 3a). However, AmB and vancomycin-loaded implants (dry and wet) showed similar hardness to the wet unloaded implant. It is worth noting the adhesiveness peak observed with both wet implants (loaded and unloaded). Regarding the manual pressure exerted on the wetted implants during different times on the surface of the acetabular component, a better adhesion was found with loaded implants, requiring 60 s to ensure excellent adhesion (Fig. 3b).

### Morphological characterization

**Fig. 4:**
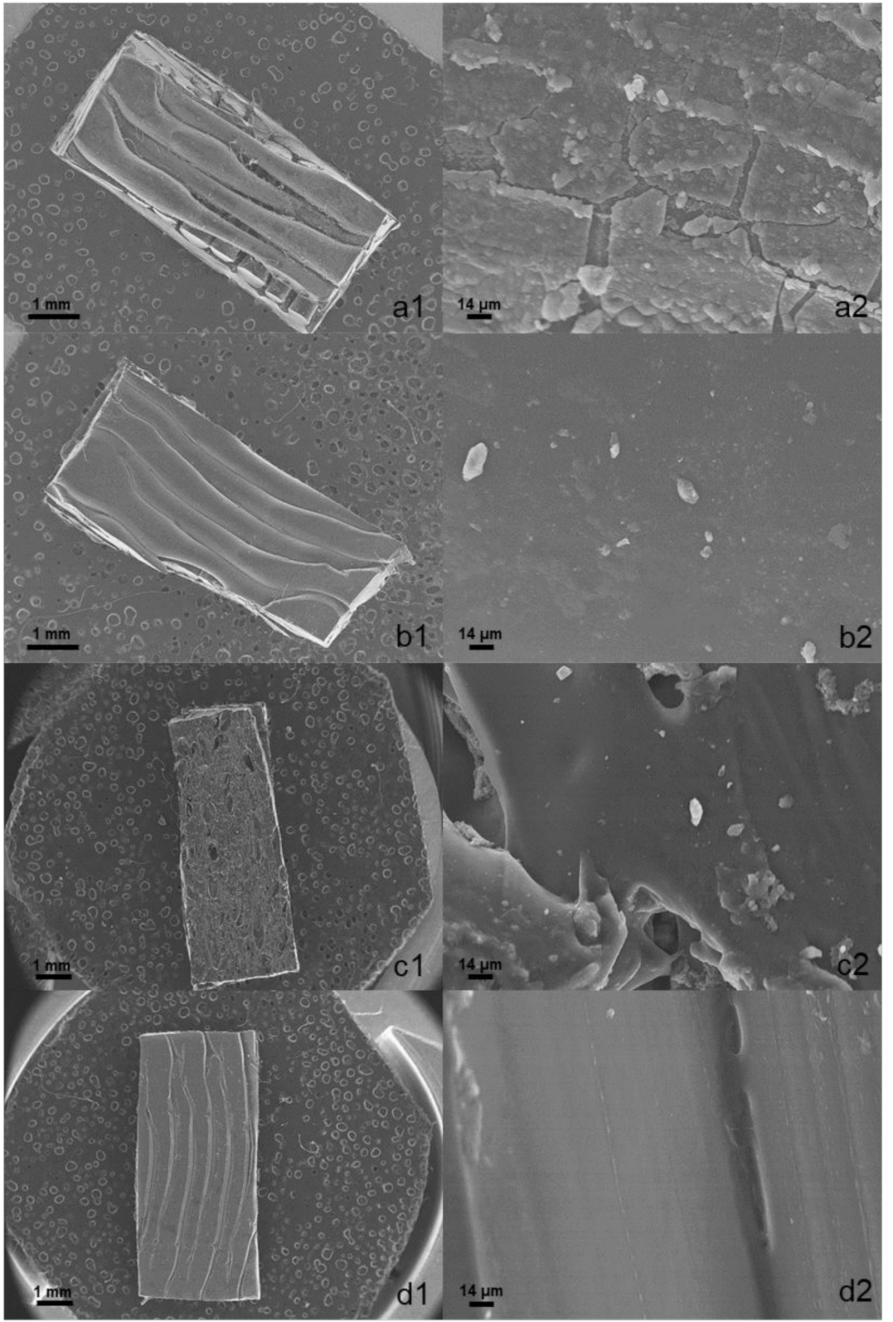
SEM micrographs of fragments of the 3DP implants. Key: (**a**) Vancomycin loaded implant at 10x (a1) and 650x (a2), (**b**) AmB loaded implant at 10x (b1) and 650x (b2), (**c**) Vancomycin and AmB loaded implant at 14x (c1) and 650x (c2), (**d**) Unloaded implant at 14x (d1) and 650x (d2)

The unloaded 3D-printed implant showed well-defined layers exhibiting a smooth surface (Fig. 4), while the loaded 3D-printed implant showed a disorganized structure indicative of partial loss of morphology after the passive diffusion process and the immersion in the solvent mixture. This corroborates the fact that longer periods of exposition (> 5 h) to further enhance drug loading are not feasible. Crystals are observed on the surface of the implant, which can be attributed to surface adsorbed drug that crystallised upon drying.

### UV sterilization

**Fig. 5:**
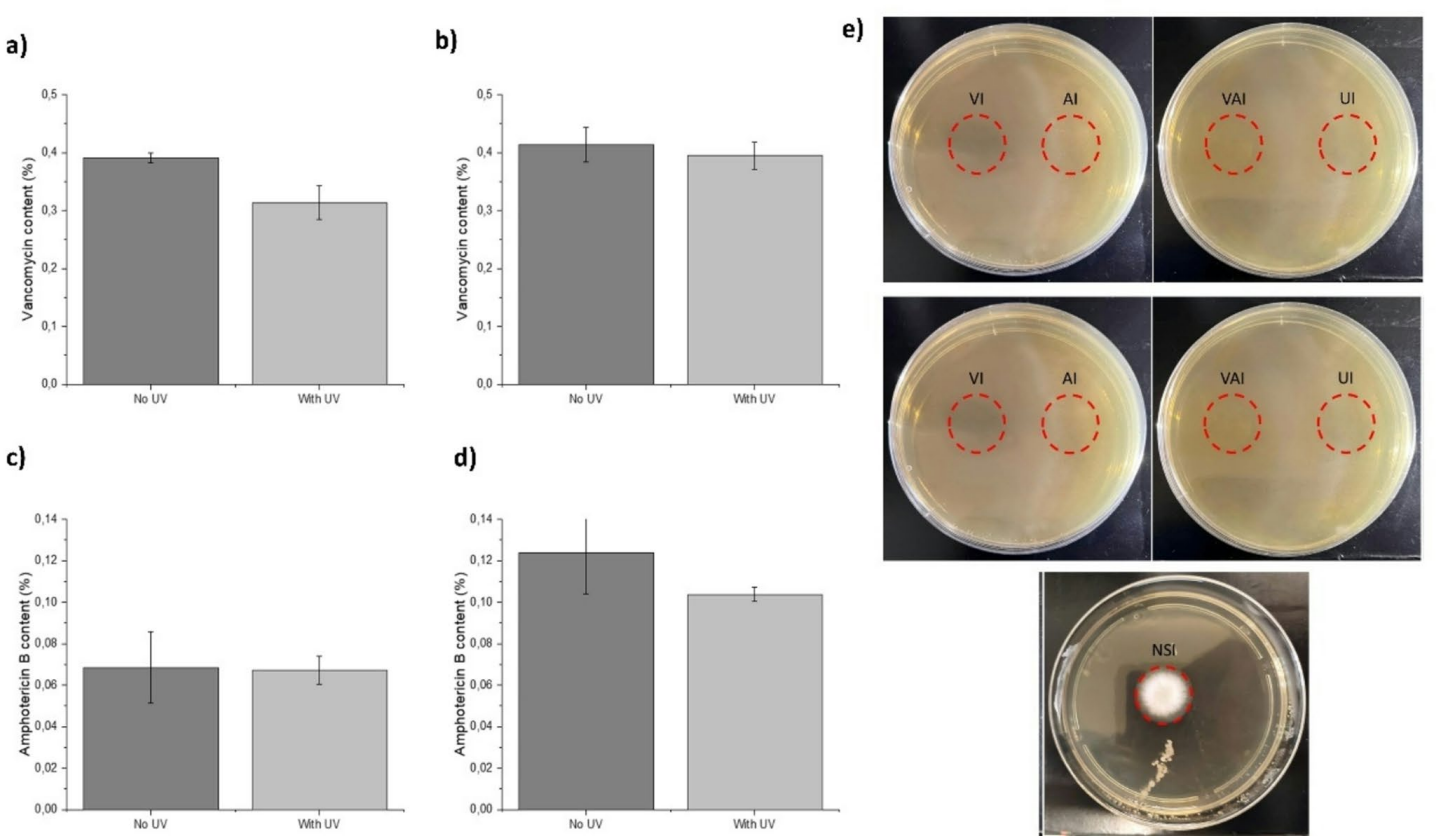
Evaluation of the drug content after implant sterilization by UV radiation. Key: (**a**) Vancomycin content in implants loaded with vancomycin and (**b**) loaded with vancomycin and AmB; (**c**) AmB content in implants loaded with AmB or (**d**) loaded with AmB and vancomycin; (**e**) MHB plates after culturing 48 h with UV sterilised loaded and unloaded 3DP implants (110 mg). Red circles indicate where the implants were placed. Key: VI, Vancomycin Implant (400 µg); AI, AmB Implant (200 µg); VAI, Vancomycin and AmB Implant (400 µg and 200 µg); UI, Unloaded Implant (control); NSI, non-sterilized unloaded implant

UV radiation led to a non-significant decrease (*p* > 0.05) in the content of both AmB and vancomycin-loaded implants (Fig. 5a-d). After 48 h, the MHB plates with the UV sterilized implants did not show any presence of fungal or bacterial growth (Fig. 4e). Based on these results, UV radiation showed to prevent microbial contamination in the implants.

### Drug release

**Fig. 6:**
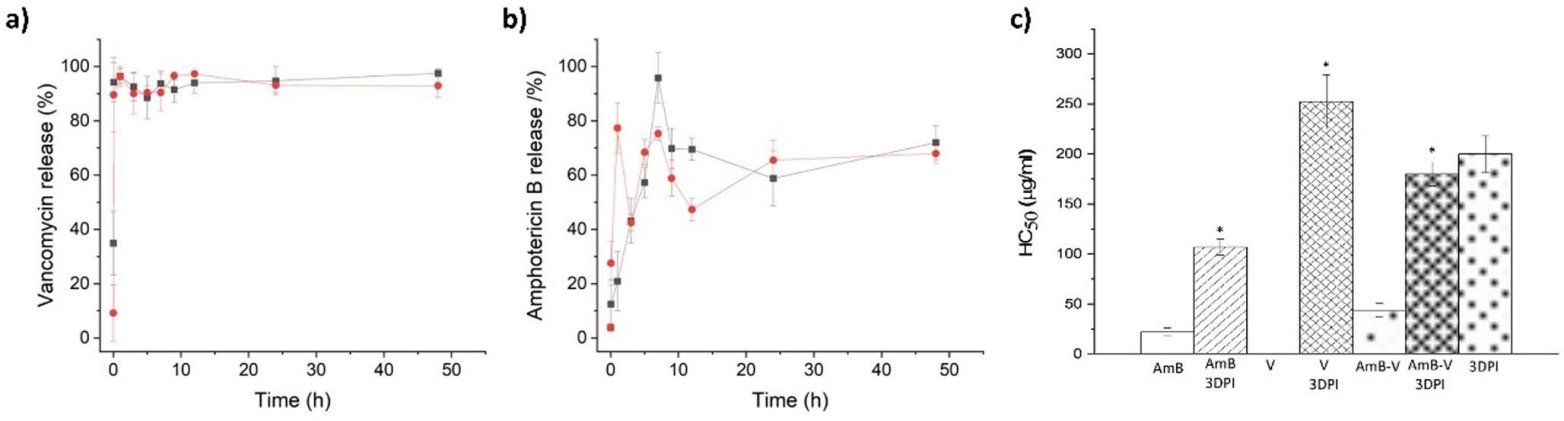
Haemolytic toxicity and antimicrobial release profile from 3DP implants adhered to the acetabular cup, with one (-■-) or both APIs (). Key: (**a**) Vancomycin release from implants loaded with vancomycin (5 mg) and vancomycin + AmB (5 mg and 2.5 mg), (**b**) AmB release from implants loaded with AmB (2.5 mg) and AmB + vancomycin (2.5 mg and 5 mg); (**c**) Haemolytic effect of the antimicrobial-loaded 3DP and the active ingredients separately and in combination. * Statistical differences (*p* < 0.05), ns (not significant). Key: V, vancomycin; AmB, Amphotericin B; 3DPI, 3D printed implant; HC_50_, concentration that causes 50% haemolysis

The release profile from both antimicrobial agents was significantly different. Vancomycin was immediately released (< 1 h) from the 3DP implant to the physiological media, which can be attributed to its hydrophilic nature and surface loading. However, AmB showed a gradual release from the 3DP implant over a 10 h period (Fig. 6a&b). No differences were observed between those implants loaded with one or two drugs. Vancomycin maintained a stable concentration in the media over a 48-hour period. In contrast, the concentration of amphotericin B (AmB) dropped significantly after 10 h. This decline is likely due to its low solubility in water, which can cause the drug to precipitate out of solution. To prevent this, albumin was added to the media to better mimic physiological conditions. Since AmB is highly bound to proteins in the body, the presence of albumin helps keep it dissolved and prevents precipitation during release.

### Haemolysis

Haemolysis was evaluated by comparing the antimicrobial-loaded 3DP implants with VAN and AmB fully dissolved either in PBS (1X, pH 7.4) or DMSO, respectively. Vancomycin did not exhibit any toxicity on RBCs at the highest tested concentration (50 µg/ml). The 3DP implant loaded with vancomycin was more haemolytic than the drug itself, but exhibited a very high HC_50_ similar to the unloaded implant, which is considered acceptable for human administration (< 5%). Nevertheless, the implant showed a significant protective effect for AmB, which is a well-known haemolytic drug [21]. The AmB 3DP implant enhanced the HC_50_ by 5-fold compared to the drug itself. A similar protective effect (4-fold HC_50_ enhancement) was observed in the combined AmB-vancomycin 3DP implant (Fig. 6c).

### Antifungal assay

**Fig. 7:**
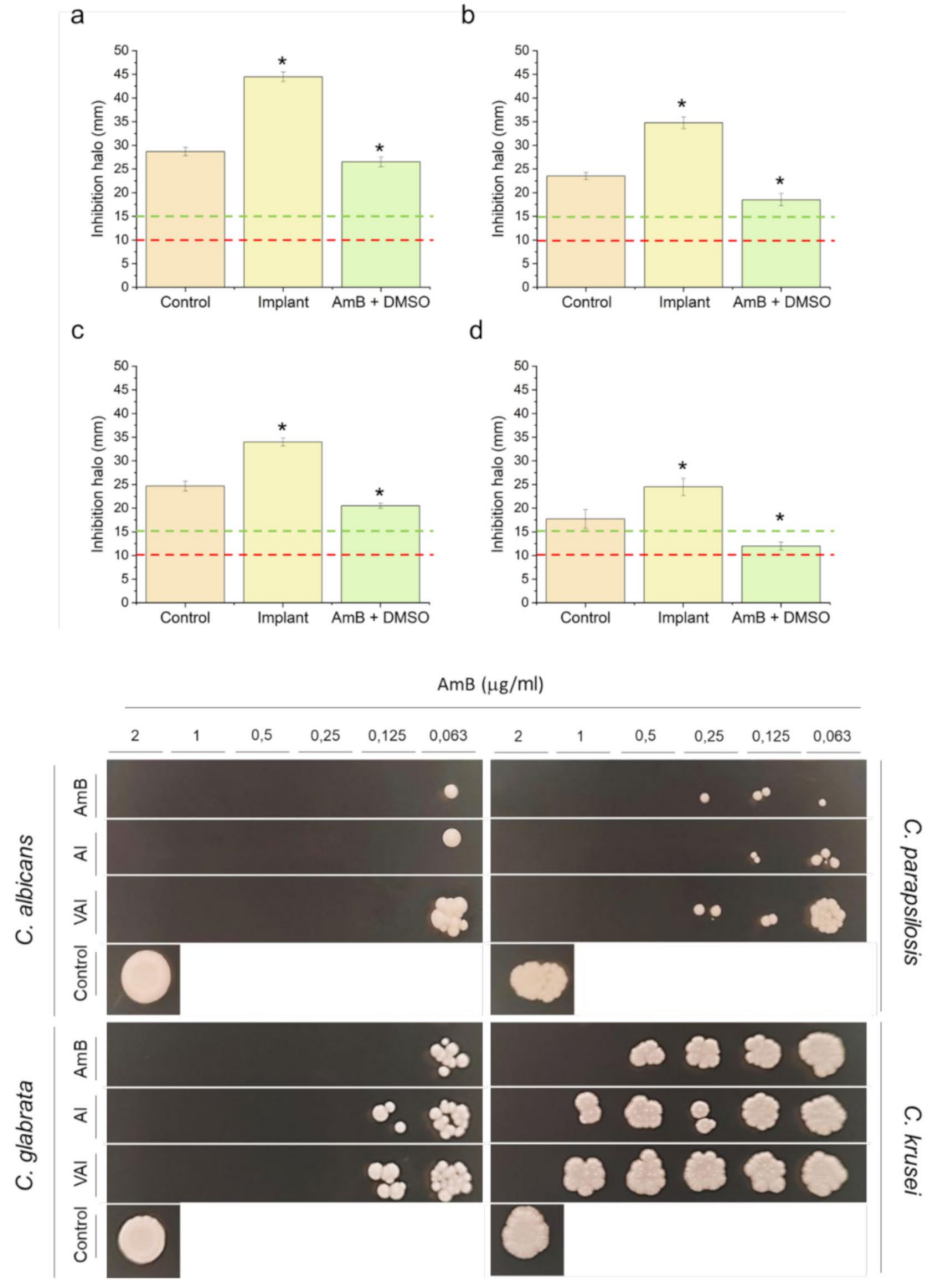
**Top panel**: In vitro antifungal activity of 3DP implants loaded with AmB against different species of Candida spp. * Statistical differences (*p* < 0.05). Key: (**a**) C. albicans; (**b**) C. parapsilosis; (**c**) C. glabrata; (**d**) C. krusei; Control, Neo-Sensitabs™ discs loaded with 10 µg of AmB; Implant, 13 mm with 200 µg of AmB; AmB + DMSO, 10 µg of AmB. **Bottom panel**: Fungal growth at different concentrations of AmB 3DP implants or vancomycin-AmB combination. Key: AI, AmB Implant, VAI, Vancomycin, and AmB Implant

*Candida albicans*,* C. parapsilosis*, and *C. glabrata* strains were susceptible to implants loaded with AmB, with an inhibition zone (halo) diameter bigger than 15 mm, which indicates that there was good diffusion of the drug from the implant into the agar. Compared to the Neo-Sensitabs™ discs loaded with 10 µg of AmB, halos of AmB dissolved in DMSO were significantly smaller, and the halos of AmB-loaded implants were significantly larger. In the case of *C. krusei*, the AmB in DMSO showed a dose-dependent efficacy against this strain as the inhibition halo was between 10 and 15 mm, indicating that a higher concentration is necessary to eradicate an infection caused by this pathogen. However, AmB implants did not show this dose-dependent profile (Fig. 7).

According to the presence or absence of colonies in Sabouraud agar plates, the MFC of AmB varied among the different species of *Candida* spp., being the most susceptible *C. albicans* with a MFC of 0.125 µg/ml followed by *C. glabrata* (0.125 µg/ml), *C. parapsilosis (*0.5 µg/ml), and *C. krusei* (1 µg/ml). AmB-loaded implants followed the same trend, but the MFC was double for *C. glabrata* and *C. krusei* (Fig. 7). The Same results were obtained for vancomycin-AmB-loaded implants. For all the species, the MIC, according to the media turbidity, was the same as the MFC. The values obtained are similar to previous studies with AmB [33–35].

### Antibacterial assay

**Fig. 8:**
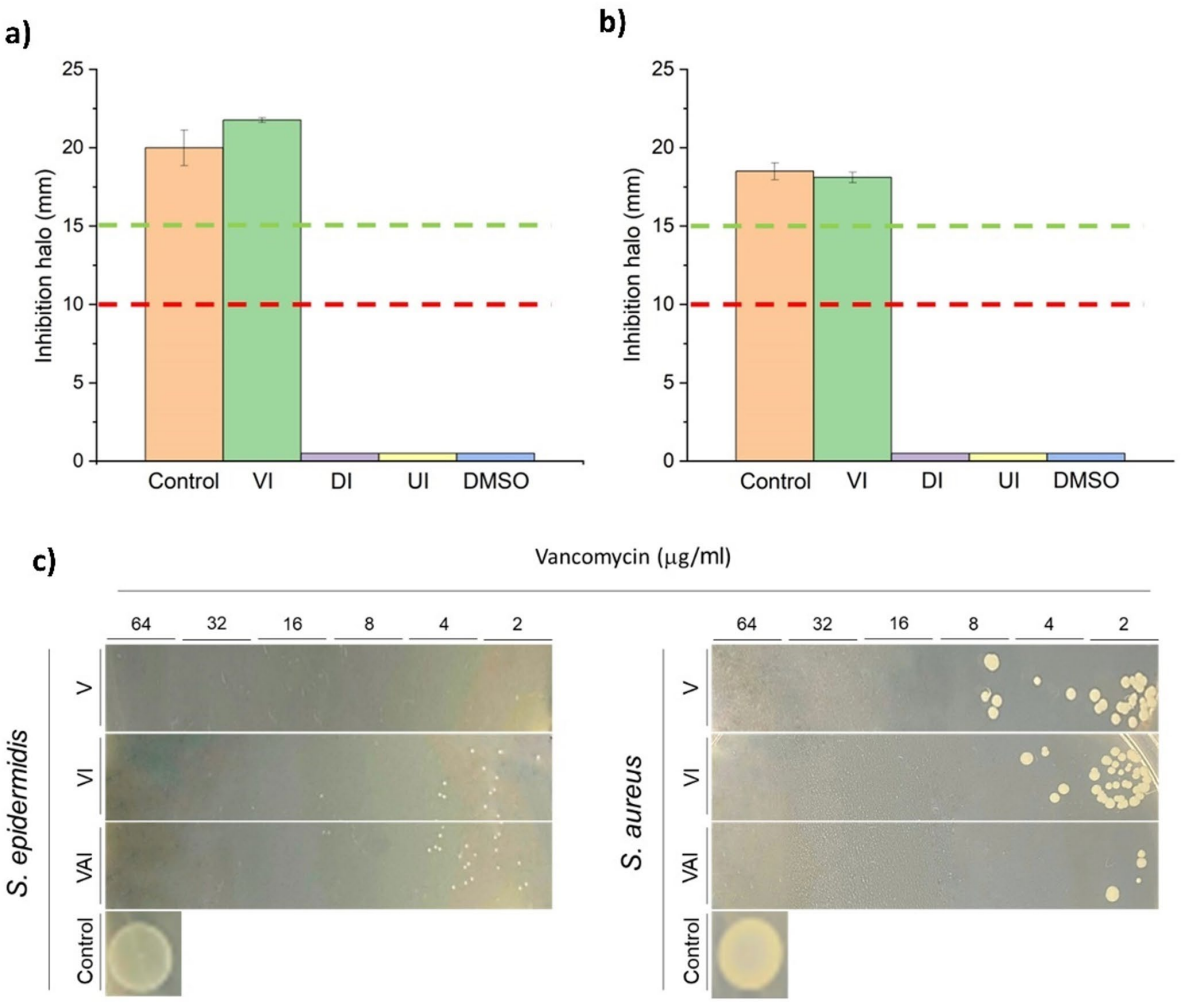
In vitro antibacterial activity of 3DP implants loaded with vancomycin against different species of Staphylococcus spp. * Statistical differences (*p* < 0.05). Key: (**a**) S. epidermidis; (**b**) S. aureus; Control, Neo-Sensitabs™ discs loaded with 30 µg of VAN; VI, implant loaded with 400 µg of VAN; DI, implant loaded with DMSO; UI, unloaded implant; DMSO, discs impregnated with DMSO; (**c**) Bacterial growth at different concentrations of vancomycin within the 3DP implants alone or in combination with AmB. Key: V, Vancomycin, VI, Vancomycin Implant, VAI, Vancomycin and AmB Implant

Both *Staphylococcus epidermidis* and *S. aureus* were susceptible to the vancomycin-loaded implants, eliciting an inhibition halo above 15 mm in diameter (Fig. 8). No significant differences (*p* > 0.05) were found between the vancomycin-loaded implant and the Neo-Sensitabs™ discs. Unloaded implants, DMSO-impregnated discs, and DMSO-loaded implants showed no inhibition halo, indicating that antibacterial activity occurs only due to the release of vancomycin from the 3DP implant.

The MIC and MBC for *S. epidermidis* were ≤ 2 µg/ml with vancomycin. However, the MIC and MBC for *S. epidermidis* were 8 µg/ml for the vancomycin-loaded 3DP implants alone and in combination with AmB. Opposite results were found for *S. aureus*. The MIC and MBC were 16 µg/ml for vancomycin, 8 µg/ml for vancomycin-loaded implants, and 4 µg/ml for vancomycin-AmB-loaded implants, indicating a synergistic effect against this microorganism (Fig. 7). The results obtained are similar to other vancomycin studies [36–38].

## Discussion

PJIs are a devastating complication of replacement surgeries, due to the elevated cost of the treatments required and the morbidity and mortality that they can lead to. The current demographic trend in developed countries is going to cause an increase in the number of arthroplasties, especially in patients with different comorbidities, which makes more urgent the need to find new strategies to approach PJIs [39].

The formation of a microbial biofilm can lead to a second surgery to remove the initial prosthesis and debride the area. This can lead to severe dysfunction of the prosthesis, impacting in patient’s quality of life as well as a high economic cost [40]. The costs associated with PJIs involve direct costs, both intra-hospital (surgery and antibiotherapy) and extra-hospital costs (rehabilitation, pharmacy, follow-up of patients), but also indirect costs such as low productivity and work absenteeism. It has been estimated that the overall cost of a hip replacement surgery without infection is 22.927€ [12.148–43.453€] while PJIs can increase the cost up to 79.188€ [39.354- 141.359€]. Depending on the country and the health system, PJIs can increase the cost by 2–4 times [41, 42]. The complexity and extended length of the treatment pathway for PJIs place a significant burden on the healthcare system, creating an unmet clinical need to find medical solutions to overcome this rising health problem [43].

The standard treatment of infection in an artificial joint is usually a two-stage process: (i) the existing joint is removed and surrounding soft tissues are cleaned out (known as debridement) and (ii) a plastic spacer and bone cement loaded with some antibiotics is placed to maintain normal joint space and alignment while the infection is treated. This procedure is followed by at least six weeks of antibiotics and the reimplantation of a new total joint arthroplasty later. Each time a joint replacement is removed, bone is lost, and tissues are damaged [44].

The use of commercial antibiotic-loaded cement made of PMMAs is used to fix the prosthesis. This antibiotic-loaded cement results in an early high local concentration of antibiotics followed by a second phase where drugs are slowly released over several months, even years, which may lead to microorganism resistance [23]. Depending on the antibiotic, in some cases, less than 10% of the added antibiotic is released from the bone cement. For example, in bone cement loaded with AmB, only a release of less than 4% of the total AmB content has been achieved [45, 46]. Since most PJIs are caused by bacteria, experience with antifungal agents is relatively scarce. AmB binds to the ergosterol on the plasma membrane of fungal cells, causing apoptosis. It is a highly effective drug, but the systemic dose intravenously administered is limited to 1 mg/kg due to its toxicity when Fungizone^®^ is used (micellar formulation) or 5 mg/kg for AmBisome^®^ (liposomal formulation) [47, 48]. Goss et al. have reported a very poor AmB release from bone cement (0.03% after one week) [49]. However, there is evidence of the human in vivo efficacy of AmB to treat bone fungal infections successfully. For example, AmB sterile powder for injection (750 mg of Fungizone^®^ commercially available formulation) was mixed with PMMA, administering a 9 mg/kg dose in a patient with osteomyelitis, which resulted in a 3.2 mg/L concentration in the surgical wound drainage after 50 h post-surgery. The patient cleared up the infection and did not suffer from any adverse effects attributed to AmB, such as infusion-related side effects (fever, chills, and rigors), nephrotoxicity, or anemia [50]. Liposomal AmB has shown better release than Fungizone^®^. However, compressive strength decreased from 67 MPa to 34 MPa after 7 days, making it unsuitable for implant fixation [51]. Similar results are achieved when mixing AmB with a pore-forming drug such as cefazolin. The antimicrobial combination within the bone cement resulted in a faster AmB release but a 2-fold decrease in compressive strength [7]. There is no way to release local AmB when an uncemented implant is used.

Even though more clinical experience has been obtained with vancomycin bone cement [52], it is still not clear which dose is more suitable to find the balance between drug elution and alteration of the compressive strength. Also, there is a large variability depending on the type of cement [53]. There is evidence that the combination of two local antibiotics in bone cement exerts a stronger and long-lasting antimicrobial effect against PJI-associated pathogens [54]. However, the potential use of combining one antibacterial with one antifungal drug has not been investigated till now.

Considering the prevalence of PJIs and the lack of suitable treatments to achieve high antimicrobial concentrations at the local site of the prosthesis, there is an unmet clinical need to find advanced medical solutions to reduce the infection rate as well as to improve the current management during secondary interventions. Previously, we have developed bioadhesive gels containing microencapsulated AmB and vancomycin for direct application on the metallic surface of the prosthesis resulting in poorer control of drug release [55]. With the flexibility that brings 3D printing, release can be tuned based on the selected materials and the chosen printing technique [56, 57].

3D printing technologies are revolutionizing the field of medicine by enabling highly customizable, patient-specific solutions that were previously unattainable with conventional manufacturing techniques. In drug delivery, 3DP has opened new avenues for the fabrication of personalized oral dosage forms with tailored drug release profiles, geometry, and dosing regimens, enhancing patient adherence and therapeutic outcomes [57–59]. Beyond pharmaceuticals, 3DP is transforming the design and production of prosthetics and implants, allowing for precise anatomical matching, improved integration with host tissues, and reduced surgical complications. These advancements are particularly beneficial in orthopedics, where complex geometries and load-bearing requirements demand high degrees of customization [60, 61]. Furthermore, 3DP has made significant strides in the development of microfluidic devices, enabling rapid prototyping and on-demand fabrication of lab-on-a-chip systems for diagnostics, organ-on-chip models, and point-of-care testing [62–64]. Collectively, these innovations underscore the transformative potential of 3DP across a broad spectrum of biomedical applications, marking a shift toward more personalized, efficient, and effective healthcare solutions.

To the best of our knowledge, this is the first report of a 3D-printed personalized implant highly adapted to the convex shape of the acetabular component that can contain one or two combined antibacterial-antifungal drugs. The design of the implant was carefully taken into consideration to ensure osteointegration, and for this reason, a bioinspired structure in a spider web was selected rather than covering the full surface area of the acetabular component, allowing the prosthesis to be in contact with the bone. The applicability of this parenteral implant can be easily extended to other types of arthroplasties, such as knee replacement. Bearing in mind the capability of 3D printing, novel designs adapted to the morphology of the prosthesis could be created and printed by trained healthcare professionals. Drug loading achieved in this work is low due to the challenges associated with FDM. AmB and vancomycin degrade above 70 °C. For this reason passive diffusion was implemented in a second step post-printing. Even though the release studies showed that clinical concentrations can be achieved in the articular volume, other techniques such as semisolid extrusion can be better to prevent drug degradation and enhance drug loading.

An additional advantage of the developed 3D-printed implants is the short implantation time below 60 s and the reduction in hardness Longer operative times have been associated with higher rates of PJIs [65, 66]. Compared to bone cement, the fixation of the implant will be much faster with a minimal impact on the overall time of the surgery. After FDM, edges of the implant may be sharp which may cause discomfort to the patient. However, after wetting, the implant surface is smoother and more malleable adjusting better to the convex surface of the metallic prosthesis. It is expected that patients remain in bed over 24 h after surgery and before exerting high mechanical forces on the implant. During this time the implant would have dissolved preventing early infections. This 3D printin g approach could be used in combination with bioadhesive gels containing antimicrobial-loaded microparticles to enhance efficacyn, especially when the gap remaining is extremely small, such as for the femoral stem [67].

PJIs are mainly caused by bacteria, being *Staphylococcus* spp. being one of the most common species [68, 69]. Fungal PJIs are less frequent, around 1%, but are the ones that present more complications and risks to the patients [13, 14]. In this scenario, a combined treatment of antibiotics and antifungals would be the best option for covering the entire PJI spectrum. One limitation of the implant is the poor drug loading achieved by passive diffusion. Extrusion of drugs and excipients combined with fused deposition modeling was not successful, as drug degradation is likely, considering that both drugs degrade at temperatures below 100 degrees [25]. Nevertheless, the drug loading achieved is enough to elicit a pharmacological effect to successfully inhibit the growth of the *Candida spp.* and *Staphylococcus spp*. A synergistic effect between AmB and vancomycin was found against *S. aureus*, one of the most common pathogens responsible for PJIs. Around 65% of PJIs occur due to the biofilm formation from the bacteria of the patient localized on the skin that penetrates during the surgical procedure in the joint space. For this reason, prophylaxis is critical during the first 24 h post-surgery [40]. Based on the release profile of the implant, both drugs would have fully eluted before 48 h, actively hampering the biofilm formation in the acute phase, and the implant may not alter the compressive strength of the prosthesis as they are placed externally on the acetabular component. When the implant was dissolved, it formed a gel, helping to retain the APIs in the joint space and delaying their clearance. However, all the antimicrobial payload was released in 48 h, showing more efficient performance than the bone cement, with elution profiles below 10% in 24 h in many cases.

The implant material, a combination of PVA and PEG, is biodegradable; hence, minimal toxicity is expected in vivo [70, 71]. The material showed high biocompatibility with RBCs compared to the hemolytic nature of AmB. 3D implants loaded with AmB showed 5-fold higher protection. According to the ASTM F756-17 standard, which provides guidelines for evaluating the hemolytic properties of materials in contact with blood, hemolysis is classified based on the percentage of red blood cell lysis observed in vitro [72]. Materials that cause less than 2% hemolysis are considered non-hemolytic, indicating excellent blood compatibility. Those that induce hemolysis between 2% and 5% are classified as slightly hemolytic, suggesting a low but acceptable level of red blood cell damage. Materials causing greater than 5% hemolysis are deemed hemolytic and may pose a risk when used in blood-contacting applications. This classification is widely used to assess the safety of biomedical devices and drug delivery systems intended for clinical use. Considering that the IC_50_ concentrations achieved in vitro are much lower than those expected to use in vivo, implants can be considered safe from a hemolytic point of view. However, additional biocompatibility studies should be performed to ensure biocompatibility with bone and cartilage cells.

Also, 3D-printed implants can be sterilized using UV radiation without altering the drug content, which is a more affordable technique than gamma radiation. UV light has a low penetration through plastic and solid materials, in comparison with other techniques [73, 74]. However, the 3DP implant height is 1 mm, and each side is exposed to the light, which could explain the efficacy of this sterilization method with the implants. This could allow a faster clinical translation, bearing in mind that current printers can be placed within a laminar flow cabinet, and due to the temperatures used during printing, any possibility of microbial growth would be eradicated [61]. However, other techniques such as gamma radiation or ethylene oxide gas sterilisation should be explored as they are commonly used in clinical settings.

## Conclusions

This study presents a novel and clinically promising approach to preventing and treating PJIs through the use of personalized, 3D-printed drug-eluting implants tailored to fit the acetabular geometry of hip prostheses. Drawing inspiration from the structural elegance of a spider web, the implant design was engineered to conform seamlessly to the curved surface of the acetabular cup, promoting stability and potential osteointegration. Fabricated from a biocompatible PVA–PEG composite, the implants demonstrated excellent safety, with hemolytic toxicity five times lower than that of free amphotericin B (AmB), and adhered rapidly to prosthetic surfaces within 60 s—an advantage that could help reduce surgical time. The implants proved effective against a broad range of pathogens. Both single-agent and dual-drug formulations exhibited strong antimicrobial activity, with the combination of AmB and vancomycin showing a notable synergistic effect against *Staphylococcus aureus*. Vancomycin was rapidly released, offering immediate protection, while AmB followed a sustained release profile, maintaining therapeutic levels for up to 10 h. Importantly, both agents retained saturation solubility for at least 48 h, effectively covering the critical early postoperative period when infection risk is highest. Together, these findings position this 3D-printed platform as a flexible, targeted, and efficient strategy for PJI management. While in vitro results are encouraging, future in vivo studies will be essential to confirm the implants’ long-term safety, efficacy, and integration within clinical workflows.

## Supplementary Information

Below is the link to the electronic supplementary material.

Supplementary Material 1

## Data Availability

All data supporting the findings of this study are available within the paper and its supplementary information files.
