# Supplementary material for: Antimicrobial 3D printed implants for periprosthetic joint infections
Source: Drug Deliv Transl Res. 2025 Aug 14;16(5):1333–48. doi: 10.1007/s13346-025-01934-5 (PMC13038732; doi:10.1007/s13346-025-01934-5)
Supplement: Supplementary file 1 — Supplementary Material 1 [file 13346_2025_1934_MOESM1_ESM.docx]

**Supplementary material**

**Antimicrobial 3D Printed Implants for Periprosthetic Joint Infections**

Iván Yuste ^a#^, Francis C. Luciano ^a#^, Carmina Rodríguez ^b^, Bianca I. Ramirez^a^, Chrysi Rapti^a^, Brayan J. Anaya^a^, Aikaterini Lalatsa^c^, Almudena Ribed-Sánchez^d^, Pablo Sanz-Ruiz^e,f^, Elena González-Burgos ^g*^, Dolores R. Serrano ^a,h*^

^a^ Pharmaceutics and Food Technology Department, Faculty of Pharmacy, Universidad Complutense de Madrid, Plaza Ramón y Cajal s/n, 28040 Madrid, Spain.

^b^ Department of Microbiology and Parasitology, Faculty of Pharmacy, Universidad Complutense de Madrid (UCM), Madrid, Spain.

^c^School of Pharmacy and Biomedical Sciences, Robertson Wing, University of Strathclyde, 161, Cathedral Street, Glasgow, G4 0RE, Scotland, UK

^d^Hospital Pharmacy Unit, Hospital General Universitario, Gregorio Marañón, Doctor Esquerdo 46, 28029, Madrid, Spain.

^e^Orthopaedic and Trauma Department, Hospital General Universitario, Gregorio Marañón, Doctor Esquerdo 46, 28029, Madrid, Spain.

^f^Department of Surgery, Faculty of Medicine, Universidad Complutense de Madrid (UCM), Madrid, Spain

^g^Department of Pharmacology, Pharmacognosy and Botany, Faculty of Pharmacy, Universidad Complutense de Madrid (UCM), Madrid, Spain.

^h^Instituto Universitario de Farmacia Industrial, Faculty of Pharmacy, Universidad Complutense de Madrid, 28040, Madrid, Spain.

^#^ First authorship shared

*Corresponding authors

Elena Gonzalez Burgos

Email: [elenagon@ucm.es](mailto:elenagon@ucm.es)

Tel: +34 91 394 22 76

Dolores R. Serrano

Email: [drserran@ucm.es](mailto:drserran@ucm.es)

**Table S1.** Design of experiments. Factors and responses evaluated.

| Experiment | Ethanol (%) | AmB (mg/ml) | Implant Height (mm) | Drug loading AmB  (%) |
| --- | --- | --- | --- | --- |
| 1 | 75 | 5 | 1 | 0.203 |
| 2 | 75 | 2.5 | 1 | 0.165 |
| 3 | 75 | 2.5 | 2 | 0.098 |
| 4 | 85 | 2.5 | 2 | 0.029 |
| 5 | 75 | 5 | 2 | 0.213 |
| 6 | 85 | 5 | 1 | 0.105 |
| 7 | 85 | 5 | 2 | 0.036 |
| 8 | 85 | 2.5 | 1 | 0.112 |


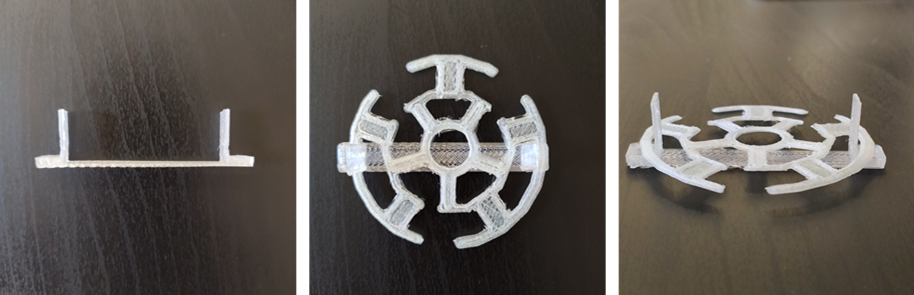


**Figure S1.** 3D printed support for holding the implant in the drug loading experiments.


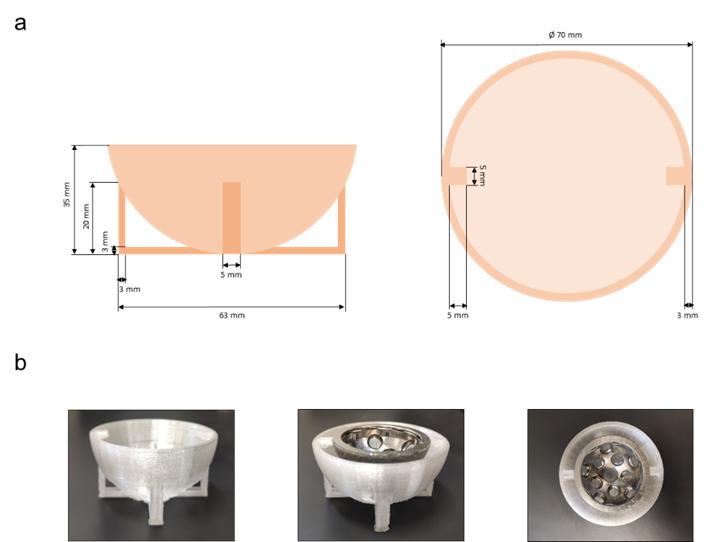


**Figure S2.** Receptacle specifically designed for the acetabular cup release experiments. Key: (a) Design and dimensions of the receptacle, front and top views, (b) Images of the 3D printed receptacle alone and with the acetabular cup.
